# MID-LONG TERM RESULTS OF MANIPULATION AND ARTHROSCOPIC RELEASE IN FROZEN SHOULDER

**DOI:** 10.1590/1413-785220172506174033

**Published:** 2017

**Authors:** HALUK CELIK, MUSTAFA FAIK SECKIN, MEHMET AKIF AKCAL, ADNAN KARA, BEKIR ERAY KILINC, SENOL AKMAN

**Affiliations:** 1. Department of Orthopedics and Traumatology, Zonguldak Ataturk State Hospital, Zonguldak, Turkey.; 2. Department of Orthopedics and Traumatology, Faculty of Medicine, Bilim University, Istanbul, Turkey.; 3. Department of Orthopedics and Traumatology, Antalya Ataturk State Hospital, Antalya, Turkey.; 4. Department of Orthopedics and Traumatology, Faculty of Medicine, Istanbul Medipol University, Istanbul, Turkey.; 5. Department of Orthopedics and Traumatology, Golhisar State Hospital, Burdur, Turkey.; 6. Department of Orthopedics and Traumatology, Florence Nightingale Hospital, Istanbul, Turkey.

**Keywords:** Bursitis/physiopathology. Bursitis/surgery. Bursitis/therapy. Joint capsule release. Manipulation, orthopedic/methods., Bursite/fisiopatologia, Bursite/cirurgia, Bursite/terapia, Liberação da cápsula articular, Manipulação ortopédica/métodos.

## Abstract

**Objective:**

Surgical treatment options should be discussed in cases of frozen shoulder, which is usually treated in a conservative manner. In this study, we evaluated the efficacy of manipulation and arthroscopic release in cases of frozen shoulder which resisted conservative treatment.

**Methods:**

A total of 32 patients who underwent manipulation and arthroscopic capsular release in 34 shoulders were included in the study. The average follow-up period was 49.5 months (range: 24-90 months). No reason for onset could be found in 8 (25%) patients, who were classified as primary frozen shoulder; twenty-four (75%) patients were classified as secondary frozen shoulder due to underlying pathologies. The average pre-operative complaint period was 11 months (range: 3-24 months). After arthroscopic examination, manipulation was performed first, followed by arthroscopic capsular release. The range of motion in both shoulders was compared before the procedure and in the last follow-up visit. Constant and Oxford classifications were used to assess functional results, and the results were assessed statistically.

**Results:**

Patient values for passive elevation, abduction, adduction-external rotation, abduction-external rotation, and abduction-internal rotation increased in a statistically significant manner between the preoperative assessment and follow-up evaluation (p<0.01). The average change of 47.97±21.03 units observed in the patients’ values obtained in the control measurements against the pre-op Constant scores was determined to be statistically significant (p<0.01). According to the Oxford classification, 29 shoulders were sufficient.

**Conclusion:**

Successful results can be obtained with arthroscopic release performed after manipulation in patients with frozen shoulder resistant to conservative treatment. **Level of Evidence IV, Case Series.**

## RESUMO

**Objetivo:**

As opções de tratamento cirúrgico devem ser discutidas nos casos de ombro congelado que, em geral, são tratadas de modo conservador. Neste estudo, avaliamos a eficácia da manipulação e da liberação artroscópica nos casos de ombro congelado refratário ao tratamento conservador.

**Métodos:**

Um total de 32 pacientes submetidos a manipulação e liberação capsular artroscópica em 34 ombros foram incluídos no estudo. O período médio de acompanhamento foi de 49,5 meses (faixa: 24 a 90 meses). Não foi possível determinar o motivo do início da afecção em 8 (25%) pacientes, que foram classificados como ombro congelado primário; 24 (75%) pacientes foram classificados como ombro congelado secundário, devido a patologias subjacentes. O período médio de queixa pré-operatória foi de 11 meses (faixa: 3 a 24 meses). Depois do exame artroscópico, realizou-se manipulação, seguida por liberação capsular artroscópica. A amplitude de movimento em ambos os ombros foi comparada antes do procedimento e na última visita de acompanhamento. As classificações de Constant e Oxford foram usadas para avaliar os resultados funcionais, e os resultados foram avaliados estatisticamente.

**Resultados:**

Os valores dos pacientes para elevação, abdução, adução-rotação externa, abdução-rotação externa e abdução-rotação interna aumentaram de modo estatisticamente significante entre a avaliação pré-operatória e a do acompanhamento (p < 0,01). A mudança média de 47,97 ± 21,03 unidades observada nos valores dos pacientes, obtidos nas medidas de controle com relação aos escores de Constant no pré-operatório foi determinada como estatisticamente significante (p < 0,01). De acordo com a classificação de Oxford, 29 ombros foram suficientes.

**Conclusão:**

Os resultados bem-sucedidos podem ser atingidos com liberação artroscópica realizada depois da manipulação dos pacientes com ombro congelado, resistentes ao tratamento conservador. **Nível de Evidência IV, Série de Casos.**

## INTRODUCTION

Frozen shoulder (FS) is a common reason for shoulder pain and loss of function. It is characterized by active and passive restriction of glenohumeral motion following frequent shoulder pain, with spontaneous onset.^1^ It was first defined by Duplay in 1872 as ‘scapulohumeral periarthritis’.^2^ The American Shoulder and Elbow Surgeons Union (ASES) defines adhesive capsulitis as a condition which occurs without the presence of a known shoulder disease, without clear etiology, in which shoulder movements are actively and passively limited at a significant level.^3^ Manipulation, arthroscopic release, or both may be performed in cases in which conservative treatment has not been successful. There is no consensus on whether manipulation should be done before or after arthroscopic release.^4^ ^-^ ^6^ In this study, we investigated the efficacy of manipulation and arthroscopic release in patients with cases of frozen shoulder which resisted conservative treatment.

## MATERIALS AND METHODS

We retrospectively evaluated patients who underwent manipulation and arthroscopic capsular release surgery to treat a diagnosis of frozen shoulder between January 2005 and July 2012. All patients in the study signed an informed consent form (Protocol number: BDH17-12-C).

We considered FS to mean active and passive range of motion (ROM) in least at two planes accompanied by shoulder pain. Criteria for inclusion in the study were existence of unilateral or bilateral FS, unsuccessful conservative treatment for at least six months, and a follow-up period of at least 24 months. Patients who had stiffness after trauma, fracture treatment, or shoulder surgery, as well as patients whose disease was associated with a non-joint pathology, were excluded from the study.

We investigated the patients’ complaints, onset of complaints, and time of first admission, and previous treatments (if any) in detail. Any concomitant systemic diseases, if present, were recorded. The patients were classified according to the method developed by Lundberg, which is based on asking whether there is an onset factor.^7^ ^,^ ^8^ According to this method, patients who did not have any factors that caused onset, any abnormal findings other than restriction of motion in the x-ray and examination, and who had an idiopathic condition were considered to have primary frozen shoulder. Patients who had a known intrinsic, extrinsic, or systemic pathology were classified as secondary FS patients. Diseases such as diabetes mellitus, hypothyroidism, hyperthyroidism, and hypoadrenalism were considered systemic reasons; diseases such as cardiopulmonary diseases, cervical disc herniation, cerebrovascular diseases, humerus fractures, and Parkinson’s disease were considered extrinsic reasons; and pathologies such as rotator cuff tendinitis, rotator cuff tear, biceps tendinitis, calcified tendinitis, and acromioclavicular arthritis were considered intrinsic reasons. The three-stage system defined by Reeves was used during patient follow-up.^8^ ^,^ ^9^

Both shoulders were examined comparatively. Values for passive elevation towards the front side, abduction, external rotation in abduction, and internal rotation in abduction were measured for each patient using a standard goniometer. The measurement of internal rotation in adduction was recorded based on the highest point the patient could reach behind his or her back. Constant and Oxford scoring were used for functional assessment.

Patients received conservative treatment for an average of 9.5 months prior to surgery (range: 6-12 months). Surgical treatment was planned for patients who did not respond to conservative treatment or who had progressive shoulder stiffness. The disease stage was considered inflammatory in patients who had severe pain as well as restriction of motion. Because surgical treatment can cause capsule damage and restriction of motion, it was postponed until the frozen stage was reached. Since the pain was felt only at the end of range of motion, it was decided that the inflammatory stage ended and frozen stage started at this point.

### Surgical technique

**Figure 1:**
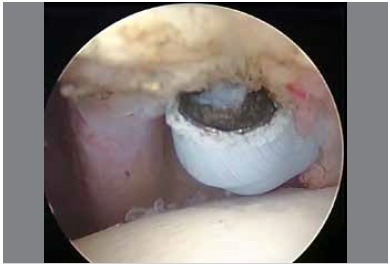
Release of rotator interval in left shoulder. Scope was inserted from posterior portal and radiofrequency probe was inserted in anterior portal.

**Figure 2:**
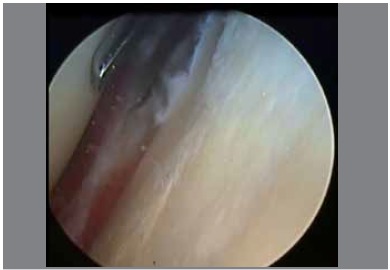
Release of posterior capsule in right shoulder. Scope was inserted from posterior portal and arthroscopic scissor was inserted in anterior portal.

All patients underwent a standardized procedure in beach-chair position. Projections of anatomic structures and entry points were marked on the skin with a pen. In order to not damage cartilage due to capsule contracture and decreased joint volume, a scope was gently inserted through the posterior portal from the head section of humerus; the joint was examined arthroscopically, and synovitis and intra-joint pathologies were recorded. Afterwards, the scope was defined and manipulation was performed. The scapula was fixed and force was gently applied through the proximal section of the humerus to elevate towards the front section and perform abduction. In patients in whom an opening was not felt, we did not proceed to the next stage. In patients in whom an opening was felt, external rotation at 0 degree abduction, external rotation in 90 degrees abduction, internal rotation in 90 degrees abduction and cross-body adduction were performed, respectively, and the manipulation was completed. After manipulation, the scope was again inserted through the posterior portal. In most cases, we observed that the anterior capsule was torn substantially. The biceps tendon was found to reach the upper border of the rotator interval. The mid-glenohumeral ligament was released from the labrum edge with a radiofrequency probe and motor trimmer inserted through a portal opened directly below the biceps tendon. The scar tissue that covered the subscapularis muscle was excised, and the subscapularis tendon was made mobile. Afterwards, the thickened scar tissue was removed from the rotator interval area of the capsule (starting from the lower edge of the biceps tendon until the upper edge of subscapularis tendon). (Figure 1) The coracohumeral ligament was separated from the coracoid process with a radiofrequency probe, and the supraspinatus tendon in the superior section, the subscapularis tendon in the inferior section, and the rotator interval section of the capsule up to the inferolateral section of the coracoid bone in the anterior section were subsequently surgically released. After release, an external rotation opening was provided in most of the patients, since the arm was on the side. The joint was examined and additional pathologies were determined. Bleeding was controlled with hypotensive anesthesia, pressurized irrigation, and radiofrequency probes. Posterior capsule release was performed in patients in whom external rotation was provided but who still did not achieve complete internal rotation, horizontal adduction, and elevation towards front section. To do so, the portals were changed on the changing rod and posterior capsule release was performed. (Figure 2) The inferior section of capsule was observed to be torn as a result of manipulation in almost all patients. Release was performed with arthroscopic scissors in patients who did not have torn structures.

A lateral portal was opened and arthroscopic subacromial bursectomy and acromioplasty were performed in patients who were considered to have subacromial compression syndrome. Afterwards, manipulation was repeated in patients who still had restricted motion. An arm sling was used after the portals were closed.

Active and passive joint motion exercises in all directions and isometric exercises were started on the first day post-operative. The patients were enrolled in a three-day intensive exercise program after the procedure, and were discharged afterward. Sutures were removed on the tenth day post-procedure, and the patients were transferred to the physical therapy and rehabilitation clinic for enrolment in a special rehabilitation program. The patients were invited for follow-up visits at week 1, week 4, month 3, month 6, and month 12 post-procedure.

NCSS (Number Cruncher Statistical System) 2007&PASS (Power Analysis and Sample Size) 2008 Statistical Software (Utah, USA) software was used for the statistical analysis. As the study data were evaluated, descriptive statistical methods (average, standard deviation, median, frequency, ratio, minimum, maximum) were used and Mann-Whitney U test was used in two-group comparisons of parameters that did not have normal distribution for comparing the quantitative data. A paired-sample T-test was used in intragroup comparison of parameters with normal distribution. The Wilcoxon signed-rank test was used in intragroup comparison of parameters without normal distribution. Significance was evaluated at p<0.01 and p<0.05 levels.

## RESULTS

A total of 32 patients with 34 shoulders were included in the study. The average age was 48 (range: 35-63). Twelve (37%) of the patients were male and 20 (63%) were female. While the right shoulder was affected in 18 patients and left shoulder was affected in 12 patients, bilateral involvement was present in two patients. The average follow-up period was 49.5 months (range: 24-90 months).

According to the Lundberg classification, no reason for onset could be found in 8 (25%) patients, and these were consequently classified as primary frozen shoulder. Twenty-four (75%) patients were classified as secondary frozen shoulder due to underlying pathologies. (Table 1) All patients received surgery during the frozen stage. The average pre-op complaint period was 11 months (range: 3-24 months).

**Table 1:** Etiologic distribution of secondary frozen shoulder.

Secondary-systemic	Secondary- extrinsic	Secondary- intrinsic
Insulin-dependent diabetes mellitus (n:7)	Coronary bypass (n:2)	Supraspinatus calcific tendinitis (n:3)
Insulin-independent diabetes mellitus (n:5)	Coronary stent (n:3)	Supraspinatus partial thickness tear (n:1)
Hyperthyroidism (n:1)	Cervical disc hernia (n:2)	

C-reactive protein and sedimentation levels were normal in all patients included in the study. Additionally, two patients had trigger finger, one patient had Dupuytren’s contracture, one patient had osteoporosis, and one patient had carpal tunnel syndrome.

The increase in the patients’ values for passive elevation towards the front, abduction, adduction-external rotation, abduction-external rotation, abduction-internal rotation measured during check-up compared to the preoperative values was statistically significant (p<0.01). (Table 2) In the preoperative evaluation, adduction internal rotation was in the hips in 12 patients, in L1 in 1 patient, in L3 in 4 patients, in the lateral thigh in 3 patients, in the L5 area in 13 patients and in T12 in 1 patient. In the follow-up evaluation, this value was in the interscapular T7 area in 21 patients, in the hips in 3 patients, in L3 in 2 patients and in the T12 area in 8 patients.

**Table 2:** Evaluation of range of motion in preoperative and follow-up periods.

(n=34)	^1^Preoperative	^2^Follow-up	Difference ^2^ ^**-**^ ^1^	Difference (%)	^**a**^ p
	Average ± SD	Average ± SD	Average ± SD	Average ± SD	
Forward elevation	86.32 ± 19.67	157.35 ± 20.20	71.03 ± 29.33	41.78 ±17.25	0.001**
Abduction	66.47 ± 25.63	151.76 ± 29.59	85.29 ± 42.16	50.17 ± 24.80	0.001**
Adduction-external rotation	21.03 ± 20.07	64.12 ± 20.61	43.09 ± 25.94	53.86 ± 32.42	0.001**
Abduction-external rotation	33.53 ± 16.86	76.47 ± 20.87	42.94 ± 24.99	47.71 ± 27.76	0.001**
Abduction-internal rotation	38.24 ± 16.51	72.79 ± 16.06	34.56 ± 19.67	38.40 ± 21.86	0.001**

^a^Paired samples t test. **p<0,01 SD: standart deviation.

The Constant score was poor in all patients before the operation. In the final follow-up visits, it was fair in 5 (15%) patients, good in 4 (12%) patients, and excellent in 25 (74%) patients. We determined that the average change of 47.97±21.03 units observed in the patients’ values obtained in the control measurements according to the pre-op Constant scores of the patients was statistically significant (p<0.01).

According to the Oxford classification used during follow-up, 1 shoulder (3%) was considered bad, 4 shoulders (12%) were considered moderate, and 29 shoulders were considered to be in sufficient condition (85%), out of a total of 34 shoulders. Three of the five patients with poor condition had diabetes (one case was bilateral). The other patient with poor condition had performed heavy lifting at work and had primary FS. These four patients stated that they could not continue their previous jobs; the remaining patients stated that they were able to continue their previous jobs and maintain their lifestyles without any problems.

No statistical difference was observed between diabetic patients and the other patients in terms of preoperative and follow-up Constant scores (p>0.05). (Table 3)

**Table 3:** Comparison of constant scores between diabetic and non-diabetic patients.

	Diabetic patients	Non-diabetic patients	*p*
	Average ± SD	Average ± SD	
Preoperative constant score	39.24 ± 5.72 (38.00)	38.23 ± 4.30 (38.00)	0.694
Follow-up constant score	91.24 ± 15.99 (95.00)	79.69 ± 25.85 (91.00)	0.066

^c^Mann Whitney U test.*p<0,05.

## DISCUSSION

The initial studies which investigated frozen shoulder, considered spontaneous remission to be absolute, and that patients could wait for recovery to occur.^10^ Although it is believed that spontaneous remission generally occurs in frozen shoulder disease, this pathological condition causes early remission in patients, and with it loss of labor and disability. Optimistic outcomes on the normal course of frozen shoulder disease have been questioned in the literature; a study published by Hand et al. with 223 patients reported that the recovery rate for this disease was 59%, complaints were still ongoing in the remaining patients, and there was functional loss in 6% of patients.^11^ During the average follow-up period of 49.5 months in our study, we observed that complaints were still ongoing in five (15%) shoulders.

Despite the fact that manipulation under anesthesia was successful in some patients, this method has been reported to cause various complications, such as humerus fracture, nerve injury, and shoulder luxation.^12^ Many successful outcomes have been reported with arthroscopic capsular release in the treatment of frozen shoulder.^13^ ^-^ ^15^ However, no agreement has been reached on the surgical technique, and opinions vary on whether it should be performed with manipulation. One study reported sufficient results in 83% of cases after arthroscopic debridement in the glenohumeral joint and subacromial area after manipulation under anesthesia, but the rate fell to 64% in the group of patients with diabetes.^16^

Another study with 26 patients evaluated anterior and anteroinferior release procedures after manipulation. No complications were reported as a result of manipulation, and poor results were obtained in three patients.^17^ Watson et al.^15^ performed arthroscopic selective release in 73 patients and followed these patients for one year; at the end of follow-up, these researchers observed that pain and restriction of motion were ongoing in 11% of the patients. Berghs et al.^18^ reported performing arthroscopic anterior and posterior capsular release in 25 patients with adhesive capsulitis, releasing the inferior capsule with manipulation; they found that the Constant score, which was 25.3 before the operation, increased to 75.5. In a similar study, arthroscopic capsule release was performed and followed by manipulation and bursoscopy in 16 patients, producing a 50-point increase in the American Shoulder and Elbow Surgeons shoulder assessment.^19^

Uhthoff and Boileau found that contractile proteins increased in the anterior capsule and rotator interval in DOH, and fibrodysplasia occurred in the posterior structures.^20^ Therefore, we also consider rotator interval release is important. In our study, we first performed an arthroscopic examination in the patients to determine existing pathologies. We then performed a gentle manipulation in an attempt to increase the range of motion. We did not observe significant complications after manipulation. We performed the manipulation before arthroscopy, contrary to other authors who performed the manipulation after arthroscopy. We believe that manipulation performed after arthroscopic capsulotomy is not effective because of fluid extravasation and swelling. We performed specific capsular release to open the range of motion in restricted directions after manipulation. We performed rotator interval release, mid-glenohumeral ligament release, coracohumeral release, and anterior and posterior capsular release, according to the direction of restriction of motion. We performed subacromial bursoscopy and bursectomy and acromioplasty in patients who were considered to have compartment syndrome and subacromial bursitis as a result of direct x-ray and magnetic resonance examinations. As a result, we observed a statistically significant increase in range of motion in all directions and Constant scores. During follow-up, good or excellent results were obtained in 29 of 34 shoulders, according to the Constant score, and sufficient result were obtained in 85% of the shoulders, according to the Oxford score.

One detail we observed in our research was outcome of surgical treatment in patients with diabetes. The relationship between frozen shoulder and diabetic patients has been mentioned in many publications; the prevalence of frozen shoulder disease is around 29-38.6% in patients with diabetes.^21^ ^,^ ^22^ The rate of occurrence is higher in patients with Type 1 diabetes than in patients with Type 2, and use of insulin and high hemoglobin A1c are among the risk factors. The risk for frozen shoulder disease increases when diabetes mellitus has been present for a long period (more than 13 years).^23^ Cınar et al.^23^ compared arthroscopic capsular release in 14 patients with diabetes and 12 patients with primary frozen shoulder, and reported that lower Constant scores were obtained in patients with diabetes. We evaluated 14 shoulders in 12 patients with diabetes in our study. We observed that the complaints were ongoing in four of the fourteen shoulders. However, no statistically significant results were obtained in the comparison of Constant scores of patients with diabetes and other patients. All of the patients who had poor results had insulin-dependent diabetes mellitus and had been using insulin for an average of 9.5 years (distribution 8-12). In addition, according to their patient history, they did not administer their insulin treatment regularly and could not obtain regular glucose regulation.

## CONCLUSION

Manipulation and arthroscopic release is an effective treatment option for frozen shoulder that resists conservative treatment. Poor results may occur in patients with insulin-dependent diabetes mellitus or treatment-resistant diabetes.
